# Antihormone treatment differentially regulates PSA secretion, PSMA expression and ^68^Ga–PSMA uptake in LNCaP cells

**DOI:** 10.1007/s00432-021-03583-w

**Published:** 2021-03-24

**Authors:** C. S. Mathy, T. Mayr, S. Kürpig, M. Meisenheimer, R. C. Dolscheid-Pommerich, B. Stoffel-Wagner, G. Kristiansen, M. Essler, M. H. Muders, R. A. Bundschuh

**Affiliations:** 1 Department of Nuclear Medicine, University Hospital Bonn, Venusberg Campus 1, 53127 Bonn, Germany; 2 Department of Pathology, University Hospital Bonn, Venusberg Campus 1, 53127 Bonn, Germany; 3 Institute of Clinical Chemistry and Clinical Pharmacology, University Hospital Bonn, Venusberg Campus 1, 53127 Bonn, Germany

**Keywords:** Abiraterone, VPC-13566, Androgen antagonist, Prostate-specific membrane antigen, [^68^Ga]Ga-PSMA-11, Prostate cancer

## Abstract

**Background:**

In recent years, a variety of innovative therapeutics for castration-resistant prostate cancer have been developed, including novel anti-androgenic drugs, such as abiraterone or VPC-13566. Therapeutic monitoring of these pharmaceuticals is performed either by measuring PSA levels in serum or by imaging. PET using PSMA ligands labeled with Fluor-18 or Gallium-68 is the most sensitive and specific imaging modality for detection of metastases in advanced prostate cancer. To date, it remains unclear how PSMA expression is modulated by anti-hormonal treatment and how it correlates with PSA secretion.

**Methods:**

We analyzed modulation of PSMA-mRNA and protein expression, ^68^Ga–PSMA uptake and regulation of PSA secretion by abiraterone or VPC-13566 in LNCaP cells in vitro.

**Results:**

We found that abiraterone and VPC-13566 upregulate PSMA protein and mRNA expression but block PSA secretion in LNCaP cells. Both anti-androgens also enhanced ^68^Ga–PSMA uptake normalized by the number of cells, whereas abiraterone and VPC-13566 reduced ^68^Ga–PSMA uptake in total LNCaP monolayers treated due to cell death.

**Conclusion:**

Our data indicate that PSA secretion and PSMA expression are differentially regulated upon anti-androgen treatment. This finding might be important for the interpretation of ^68^Ga–PSMA PET images in monitoring therapies with abiraterone and VPC-13566 in prostate cancer patients, but needs to be validated in vivo.

## Introduction

Prostate-specific membrane antigen (PSMA, also known as glutamate carboxypeptidase II, *N*-acetyl-L-aspartyl-L-glutamate peptidase I, and folate hydrolase 1) is a type II transmembrane glycoprotein acting as carboxypeptidase, highly expressed in prostate- and prostate cancer tissue (Chang 2004; Meller et al. 2015; Horoszewicz et al. 1987). Its role in prostate cancer biology is not fully understood. Biochemical studies indicate that PSMA in its dimeric form activates glutamate receptor I and, subsequently, the PI3K/Akt/mTOR1 pathway (Kaittanis et al. 2018).

Several therapeutics and molecular switches modulating androgen receptor signaling regulate PSMA expression. These include dihydrotestosterone (Wright et al. 1996; Evans et al. 2011), abiraterone (Meller et al. 2015; Murga et al. 2015), enzalutamide (Murga et al. 2015; Kranzbühler et al. 2018), and vitamin D3 (Serda et al. 2008). Interestingly, diabetes mellitus type II also modulates PSMA expression (Lutz et al. 2018). Therefore, uptake of PSMA-based tracers may be altered by intake of these drugs or under such metabolic conditions. Indeed, recent studies demonstrate increased uptake of PSMA PET tracers by tumor tissue due to treatment with dutasteride, abiraterone, enzalutamide and ARN-509 (Meller et al. 2015; Evans et al. 2011; Kranzbühler et al. 2018, 2019; Hope et al. 2017; Lückerath et al. 2018), suggesting higher detection rates.

The active site of PSMA can be targeted by small molecules mimicking glutamate binding with the help of phosphonate or urea-binding motifs (Wüstemann et al. 2019). Positron emitting diagnostic tracers like [^68^Ga]Ga-PSMA-HBED-CC ([^68^Ga]Ga-PSMA-11 (Eder et al. 2012)) and [^18^F]F-PSMA-1007 (Rahbar et al. 2018) as well as the β^−^ particle emitting radionuclide therapy tracer [^177^Lu]Lu-PSMA-617 (Benešová et al. 2015) are based on the latter and improve theranostics for CRPC patients. Prospective phase III trials in CRPC patients are currently ongoing (VISION *Trial*; NCT03511664).

PSMA PET is highly sensitive and specific in the detection of metastases and local recurrence in hormone-sensitive and castration-resistant advanced prostate cancer (Hofman et al. 2020). Therefore, PSMA PET facilitates exact restaging for therapy planning, early detection of tumor progression under therapy and assessment of total tumor burden (Ceci et al. 2015, 2019). As molecular imaging by PSMA PET is not available at many centers (Maurer et al. 2016), planning and monitoring of therapy are mostly based on changes of serum PSA levels (Wallace et al. 2014; Rao et al. 2018; Ferguson et al. 2019). Changes in serum PSA levels allow discrimination of successful treatment from failure (Ahmadzadehfar et al. 2017; Broeck et al. 2019; Mottet et al. 2020). However, PSA levels only reflect total tumor burden and do not indicate the sites of tumor progression or tumor regression (Evans et al. 2011). Therefore, it is necessary to combine PSMA PET and PSA measurement for restaging of patients with advanced prostate cancer. To date, it has not been clarified whether PSMA expression on cancer cells and secretion of PSA are regulated by the same molecular mechanisms. In particular, a better understanding of modulation of PSA expression and PSMA expression by innovative therapeutics active in prostate cancer patients resistant to hormone deprivation, such as abiraterone and 2-(7-Methyl-11-indol-3-yl)quinoline (VPC-13566), would greatly support therapy planning and monitoring under these conditions.

Abiraterone acts on the androgen-binding site and inhibits cytochrome P450 17A1 (CYP17A1) as a second way of action. Cytochrome P450 is a 17α-hydroxylase, important in the biosynthesis of dihydrotestosterone, acting on the ABS by itself (Murga et al. 2015; Soifer et al. 2012; Richards et al. 2012).

In contrast, VPC-13566 inhibits the binding function 3 (BF3) of the androgen receptor and thereby disrupts the interaction with its co-activator proteins (Lallous et al. 2016). Therefore, VPC-13566 is a promising tool when treatment with abiraterone or enzalutamide fails. Effects of inhibitors targeting BF3 on PSMA expression have not been studied to date.

Therefore, we aimed to examine the effect of VPC-13566 and abiraterone on PSMA expression at three different levels of hierarchy: (i) mRNA, (ii) total protein, (iii) uptake of PET tracer [^68^Ga]Ga-PSMA-11 in a dose- and time-dependent manner in LNCaP cells. We decided to test [^68^Ga]Ga-PSMA-11 as it is currently the most widely used PSMA-based PET tracer (Kuten et al. 2020). We also correlated PSA secretion by tumor cells under the same conditions with PSMA expression.

## Materials and methods

### Cell culture

LNCaP cells were obtained from the German Collection of Microorganisms and Cell Cultures (DSMZ, Braunschweig, Germany). Cells were grown in Dulbecco's Modified Eagle Medium (DMEM; Pan Biotech, Aidenbach, Germany) supplemented with 10% fetal bovine serum, 1% glutamine and 1% penicillin/streptomycin (all PAN-Biotech) and plasmocin (InvivoGen, San Diego, USA). PC3 prostate cancer cells were grown in Roswell Park Memorial Institute (RPMI) 1640 medium (ThermoFisher, Waltham, USA), supplemented with 10% fetal bovine serum and 1% penicillin/streptomycin (both ThermoFisher). Identity of PC3 cells in-house was verified by cell line authentication (Multiplexion, Heidelberg, Germany). Cells were incubated at 37 °C and 5% CO_2_ and seeded 96 h before further analysis of anti-androgen effects. After 24 h, a medium change was carried out and anti-hormones added to half of the cells for 72 h of treatment; 48 h after seeding, anti-hormones were added to the other half for the 48 h determination. Different concentrations of abiraterone acetate (1, 2.5, 5 and 10 μM) and VPC-13566 (1, 10, 50, 100, 200 nM and 1, 2.5, 5, 10, 15 μM) dissolved in DMSO were used (all Merck, Darmstadt, Germany). Cells incubated for 72 h with DMSO were used for control.

### Analysis of gene expression

To study mRNA expression of folate hydrolase 1 (FOLH1/PSMA) and kallikrein-3 (KLK3/PSA) in LNCaP cells under the influence of the anti-androgens abiraterone acetate and VPC-13566, cells were seeded 96 h prior to RNA isolation in fibronectin (0.02 mg/mL, AppliChem, Darmstadt, Germany) coated wells with a cellular density of about 21,000 cells/cm^2^. Antiandrogen treatment was performed as stated above. After trypsinization, the RNA was extracted using the RNeasy Plus Mini Kit (Quiagen, Hilden, Germany) as recommended by the manufacturer. Here, the samples are first lysed in a guanidine-isothiocyanate buffer, passed through a gDNA eliminator column, bound in the presence of ethanol to RNeasy spin column and the RNA finally eluted and quantified. RNA was reverse-transcribed to cDNA using recombinant reverse transcriptase (RevertAid H Minus RT), random hexamer primers and deoxynucleotide triphosphates (dNTPs) as well as a RNAse inhibitor (RiboLock, all ThermoFisher). Real-time polymerase chain reaction (qRT-PCR) was performed on a thermocycler (7500 Fast Real-Time PCR System, Applied Biosystems, Foster City, USA) with Taq DNA polymerase and monitored by intercalating the fluorescent dye SYBR Green in the generated DNA, with ROX as reference dye (Maxima SYBR Green/ROX qPCR Master Mix, ThermoFisher). The primer sequences for FOLH1, KLK3 and PPIA (peptidylprolyl isomerase A, used as housekeeping gene) were designed and the respective oligonucleotides sourced from Eurofins (Luxembourg, Luxembourg):

FOLH1 forward 5′- TTGTTTGCAAGCTGGGATGC-3′, reverse 5′-AATATAAGCCACGCCACGCT-3′; KLK3 forward 5′-GGTTGTCTTCCTCACCCTGT-3′, reverse 5′-GAATGCTTCTCGCACTCCCA-3′; PPIA forward 5′-GCTGGACCCAACACAAATGG-3′, reverse 5′-GGCCTCCACAATATTCATGCCT-3′. Sequence verification of the amplification products was performed by Sanger sequencing. Relative gene expression was assessed using the $2^{-\Delta\Delta\text{C}_{\text {t}}}$ method with PPIA as housekeeper gene and DMSO-treated LNCaP cells as reference system.

### Analysis of PSMA protein expression

Total protein expression of prostate-specific membrane antigen (PSMA) was studied via Western blotting. LNCaP cells were seeded at a cellular density of 21,000/cm^2^ in wells with anti-hormonal treatment. Subsequently, cells were lysed with radio-immunoprecipitation assay buffer (RIPA buffer, ThermoFisher) including protease and phosphatase inhibitors (Complete Protease Inhibitor Cocktail, Roche, Basel, Switzerland and Halt Protease and Phosphatase Inhibitor, ThermoFisher). Protein amounts were quantified with the BCA protein assay kit (ThermoFisher), bovine serum albumin served as standard. For every anti-hormone treatment condition, 15 µg of protein lysate was separated by SDS polyacrylamide gel electrophoresis and transferred to PVDF membranes. Membranes were blocked and incubated with primary antibodies against PSMA (M3620, mouse monoclonal, dilution 1/1000, Dako/Agilent, Santa Clara, USA) and for protein loading control, against TATA-binding protein (1TBP18, mouse monoclonal, dilution 1/2000, abcam, Cambridge, UK). Primary antibody binding was visualized by incubation with secondary anti-mouse antibodies (ab6789, goat polyclonal, dilution 1/10,000, abcam) and subsequent application of luminol-based chemiluminescent substrates.

Chemiluminescence was detected with the Fusion Solo S Bioimaging System (Vilber, Eberhardzell, Germany) and intensities quantified using ImageJ (NIH, Beteshda, USA). Intensity values were calculated for PSMA as summation over both bands and divided by the associated TBP band. Data were normalized to DMSO treatment and the values of the respective anti-hormone treatments related thereto.

### Secretion of PSA by LNCaP cells

The secretion of the prostate-specific antigen (PSA/KLK3) by LNCaP cells under anti-androgen therapy was tested in the cell supernatants of the other series of experiments before trypsinization, before lysis or before incubation with [^68^Ga]Ga-PSMA-11, i.e. 72 h or 48 h, respectively, after onset of the anti-hormone treatment. Electro-chemi-luminescence method (ECLIA) was used to determine total PSA (free and complexed), the same method as applied in routine clinical diagnostics on human serum samples (Haese et al. 2002). Measurements were carried out after centrifugation of the samples according to the Elecsys total PSA protocol, using the cobas e TPSA reagents on the cobas e 801 automated analyzer according to the manufacturer's instructions (Roche Diagnostics, Rotkreuz, Switzerland) (Blackburn et al. 1991; Marquette and Blum 2008). PSA concentrations are determined by a calibration curve (Reference Standard WHO 96/670 90% PSA-ACT+10% free PSA).

### Synthesis of [^68^Ga]Ga-PSMA-11

First synthesis of PET tracer [^68^Ga]Ga-PSMA-11 ([^68^Ga]Ga-Glu-NH-CO-NH-Lys(Ahx)-HBED-CC) was described by Eder et al*.* (2012). Labeling of PSMA-11 with Gallium-68 in this study was carried out according to the manufacturer's instructions as specified previously on an automate-cassette module (GAIA, Elysia-Raytest, Straubenhardt, Germany) (Meisenheimer et al. 2019). 50 µg PSMA-11 was radiolabeled with 500 µL ^68^Ga-eluate obtained from a ^68^Ge/^68^Ga-generator (iThemba LABS, Somerset West, South Africa) in 3.6 mL 0.08 M ammonium acetate buffer at pH 4.5 (at 95 °C for 6 min). Specific activities of 330 ± 4 MBq/nmol were achieved. Reaction product was diluted with ≈ 5 mL water and then purified using a C18 column. Subsequently, the product was eluted with 60% ethanol, the cartridge rinsed with 8.5 mL saline and sterile-filtered for final formulation. Radioactivity and half-life were measured with a dose calibrator (ISOMED 2010, MED-Nuklearmedizintechnik, Dresden, Germany). Chemicals and peptide were purchased from ABX (Radeberg, Germany). The final product was further diluted with cell medium including supplements to a radioactive concentration of 3.7 MBq/mL and immediately employed for the uptake experiments. Quality control with regard to yield, unbound [^68^Ga]Ga^3+^ and colloid content was performed with the help of thin-layer chromatography (TLC) and high-pressure liquid chromatography (HPLC).

### Uptake of [^68^Ga]Ga-PSMA-11 into LNCaP cells

For the uptake measurements, cells were seeded with a density of 20,725 cells/cm^2^ in fibronectin (0.02 mg/mL, AppliChem) coated wells and treated as stated above. Furthermore, additional wells were prepared without cells to exclude subsequent unspecific binding to the well plate and cell media components, which is, however, almost negligible. Next, cell media were changed prior to addition of [^68^Ga]Ga-PSMA-11 with a final activity in a well of 370 kBq/mL. Incubation for 90 min at 37 °C and 5% CO_2_ was terminated by ice-cold PBS, followed by washing twice with PBS (PAN-Biotech). Cells were trypsinized (Trypsin/EDTA, PAN-Biotech) at 37 °C and 5% CO_2_. Process was stopped with cell medium after 5 min. 60 µL of the total volume was kept with a cassette for cell counting via an automated cell counter according to manufacturer’s instructions (Via1-Cassette™, NucleoCounter® NC-200™, Chemometec, Allerod, Denmark). The wells were further washed twice with 2% Triton™ X-100 and the resulting fractions were measured together with the volume remaining from trypsinization using a γ-counter (WizardTM3'', Perkin Elmer-Wallac, Shelton CT, USA). To obtain the maximum possible uptake, the added [^68^Ga]Ga-PSMA-11 alone was measured without further manipulation. The measured activities were converted by the device into counts per minute and indicated together with the time of measurement. To obtain the total uptake, the measured values were corrected for half-life and normalized to a measurement time and the values of the measured maximum possible uptake. For proliferation-independent values, the total uptake values were related to the cell numbers (normalized to 10^5^ cells).

### Data analysis and statistical evaluation

Data analysis was performed with Excel (version 12.0, 2007, Microsoft, Redmond, USA) except for sigmoidal curve fitting. The adjustment of Boltzmann sigmoid functions was carried out via a scaled, Levenberg–Marquardt algorithm (SciDAVis, version 1.23, 2018, Free Software Foundation, Boston, USA). Mean values were compared by Student’s *t* test where appropriate (SPSS, version 25, 2017, IBM, Armonk, USA), one-sided tests when PSMA increase is hypothesized, two-sided tests in all other cases. Pearson correlation coefficients were calculated using SPSS. *p* values ≤ 0.05 were considered significant. Data are presented as means ± standard error of the mean (± SEM).

## Results

**Fig. 1:**
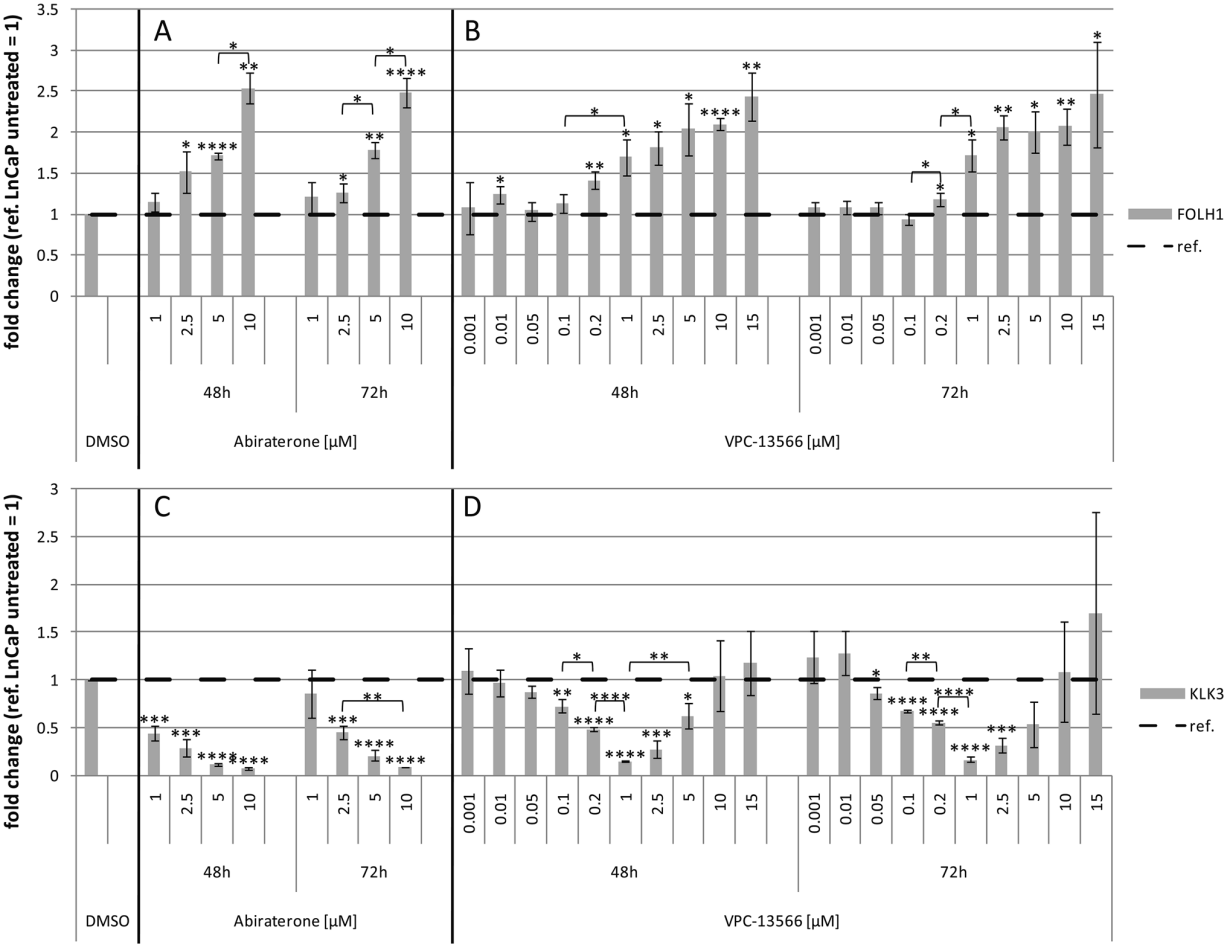
mRNA expression of **a**, **b** FOLH1 (PSMA) and **c**, **d** KLK3 (PSA) in LnCaP- cells treated with abiraterone or VPC-13566. mRNA expression of **a**, **b** FOLH1 (PSMA) and **c**, **d** KLK3 (PSA) in LnCaP cells treated for 48 h, respectively, 72 h with either DMSO (ref.: = 1) or increasing concentrations of antihormons **a**, **c** abiraterone acetate (1, 2.5, 5 and 10 µM) or **b**, **d** VPC-13566 (1, 10, 50, 100, 200 nM and 1, 2.5, 5, 10, 15 µM for VPC-13566). Values presented are mean values, depicted errors standard error of mean of *n* = 3 experiments. *: = *p* < 0.05, **: = *p* < 0.01, ***: = *p* < 0.001, ****: = *p* < 0.0001

We investigated the effect of abiraterone or VPC-13566 on PSMA-mRNA, and on expression of total protein and surface protein in LNCaP cells. In parallel, we determined RNA expression and protein secretion of PSA. Treatment with abiraterone or VPC-13566 for different periods of time (48 h and 72 h) induced upregulation of PSMA (FOLH1) mRNA in a dose-dependent manner (Fig. 1a and b). In contrast, abiraterone or VPC-13566 treatment reduced the expression of PSA (KLK3) in a concentration-dependent manner (Fig. 1c). Interestingly, differences in the maximum reduction between abiraterone and VPC-13566 were not significant (*p* = 0.089), whereas VPC-13566 showed the effect at a tenfold lower concentration. Moreover, VPC-13566 concentrations higher than 2.5 µM were less active and concentrations higher than 10 µM did not reduce PSA expression (Fig. 1d). Incubation with 1 µM VPC-13566 over 48 h resulted in a reduction to 0.153 ± 0.004, whereas incubation with 5 µM leads only to a reduction to 0.632 ± 0.129 (*p* = 0.008). Effects were independent of incubation times (e.g. VPC-13566 48 h, 1 µM vs. VPC-13566 72 h, 1 µM, *p* = 0.570). Comparing PSMA with PSA expression revealed a significant negative correlation by abiraterone treatment over the entire measured concentration range (Pearson correlation coefficient *ρ* = − 0.745, *p* = 0.0014) as well as for VPC-13566 between 1 nM and 1 µM (*ρ* = − 0.457, *p* = 0.028).

**Fig. 2:**
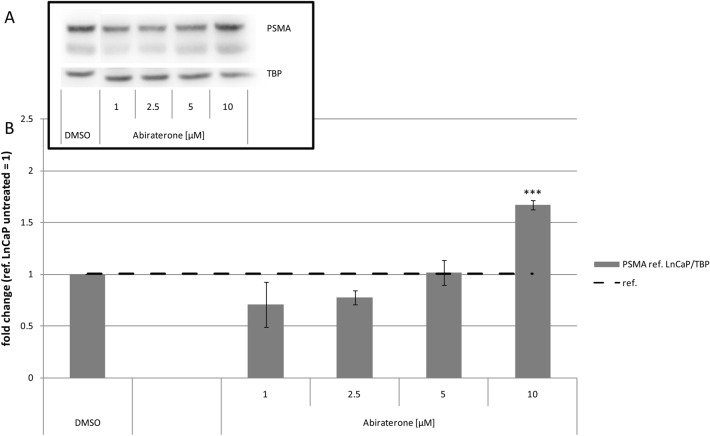
Protein expression of PSMA in LnCaP cells treated with abiraterone. **a** Total protein expression of PSMA determined via Immunoblotting in LnCaP cells treated for 72 h with either DMSO (ref.: = 1) or increasing concentrations of anti-hormone abiraterone acetate (1, 2.5, 5, 10 µM), reference TBP. **b** Protein quantification. Values presented are mean values, depicted errors standard error of mean of *n* = 3 experiments. ***: = *p* < 0.001

**Fig. 3:**
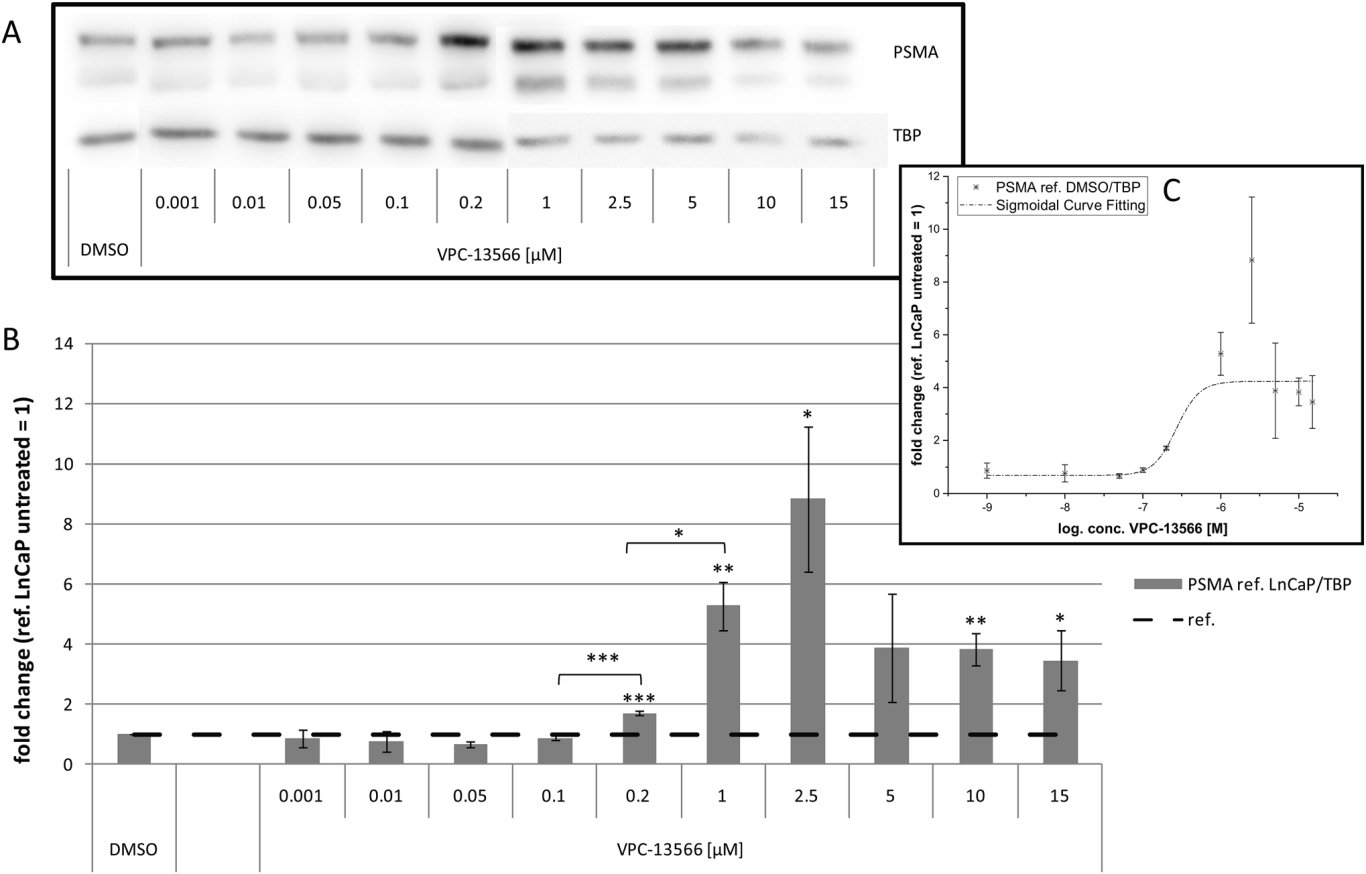
Protein expression of PSMA in LnCaP cells treated with VPC-13566. **a** Total protein expression of PSMA determined via Immunoblotting in LnCaP cells treated for 72 h with either DMSO or increasing concentrations of anti-hormone VPC-13566 (1, 10, 50, 100, 200 nM and 1, 2.5, 5, 10, 15 µM), reference TBP. **b** Protein quantification. Values presented are mean values, depicted errors standard error of mean of* n* = 3 experiments, normalized to TBP. *: = *p* < 0.05, **: = *p* < 0.01, ***: = *p* < 0.001. **c** Sigmoidal curve fitting

In a second step, we compared the effect of abiraterone or VPC-13566 on PSMA protein expression by Western blotting. Regardless of the anti-hormone treatment, PSMA double bands were detected belonging most probably to different glycoforms of PSMA as previously reported (Murga et al. 2015; Israeli et al. 1993). The antihormonal treatment did not change the relationship of the two variants to each other. Incubation with 10 µM abiraterone led to a significant increase of total PSMA protein levels (1.67 ± 0.05-fold, *p* = 0.0001) compared to control cells (Fig. 2). Lower concentrations of abiraterone did not exhibit significant values. Treatment with VPC-13566 (Fig. 3) resulted in a significant concentration-dependent increase of the total PSMA protein expression with a maximum at 2.5 µM (8.83 ± 2.39, *p* = 0.015). Correlation analysis indicates that the dose–response curve shows a sigmoidal shape (*R*^2^ = 0.959) with an EC_50_ value of 271 ± 33 nM (Fig. 3c).

**Fig. 4:**
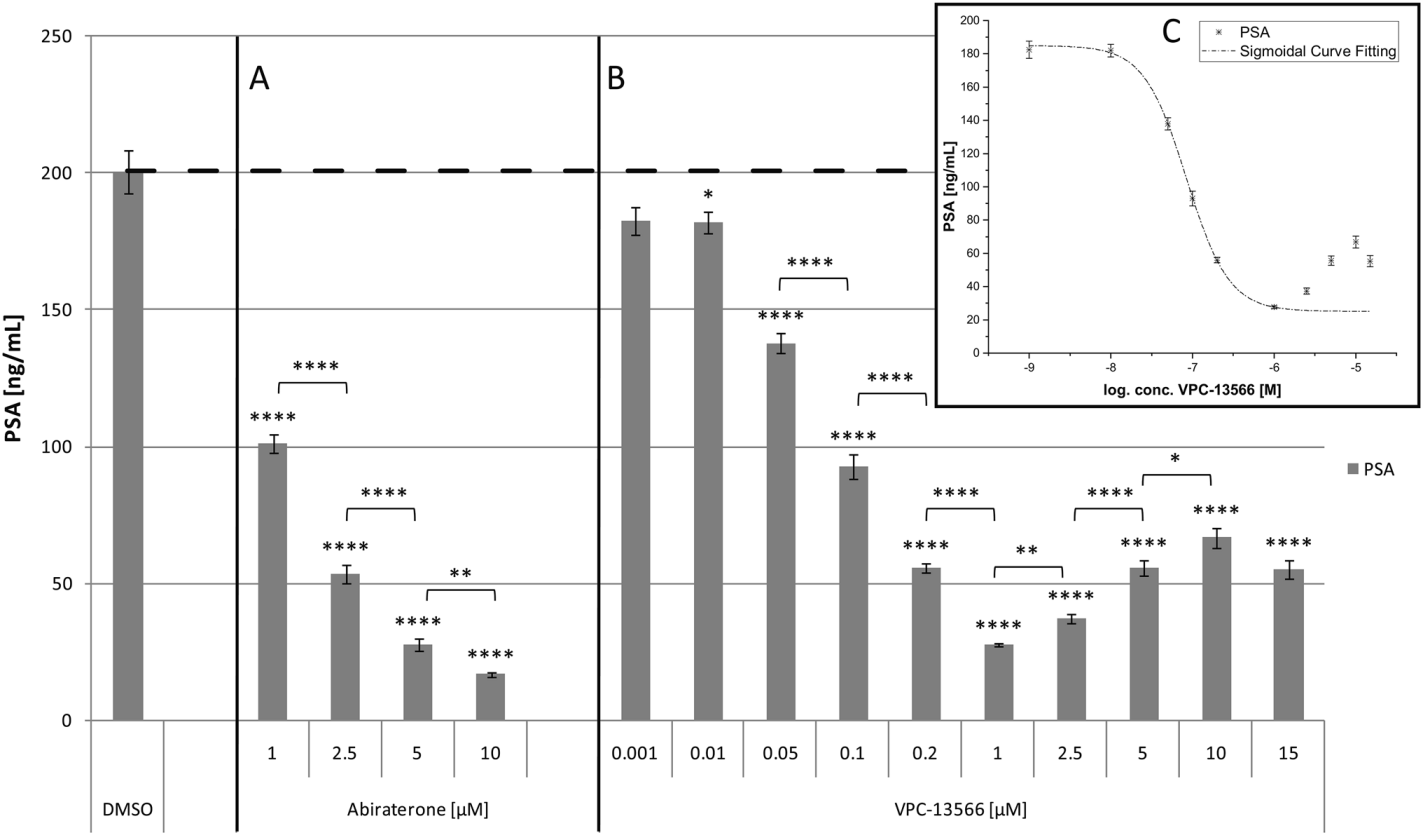
PSA secretion of LnCaP cells treated with abiraterone or VPC-13566. PSA secretion determined via ECLIA of LnCaP cells treated for 72 h with either DMSO or increasing concentrations of anti-hormones **a** abiraterone acetate or **b** VPC-13566 (1, 2.5, 5 and 10 µM for abiraterone acetate and 1, 10, 50, 100, 200 nM and 1, 2.5, 5, 10, 15 µM for VPC-13566). Values presented are mean values, depicted errors standard error of mean of n = 6 experiments. *: = *p* < 0.05, **: = *p* < 0.01, ****: = *p* < 0.0001. **c** Sigmoidal curve fitting for VPC-13566 in the concentration range up to 1 µM

Next, we determined PSA protein secretion into culture supernatants by LNCaP cells after treatment with abiraterone or VPC-13566 using ECLIA. Abiraterone induced a concentration-dependent decrease of PSA secretion. PSA concentration was 16.9 ± 0.9 ng/mL at the highest anti-hormone concentration after 72 h, compared to 200.7 ± 7.9 ng/mL in control cells (*p* = 3.04 × 10^–9^), consistent with the decrease in gene expression in the same concentration range (Fig. 4a). VPC-13566 (Fig. 4b) also showed a strong decrease in PSA concentration with an increase in anti-hormone concentrations after 72 h. The strongest effect was seen with 1 µM VPC-13566, where a reduction to 27.6 ± 0.8 ng/mL was seen (*p* = 5.39 × 10^–9^). Above 1 µM, the effect diminished significantly (*p* = 0.001), but in contrast to gene expression, some effect was still present at 10 µM with a reduction to 66.9 ± 3.6 ng/mL (*p* = 1.14 × 10^–7^). The dose–response relation matches a sigmoidal curve (*R*^2^ = 0.999879) in the concentration range up to 1 µM. IC_50_ value was 83.2 ± 5.8 nM (Fig. 4c).

**Fig. 5:**
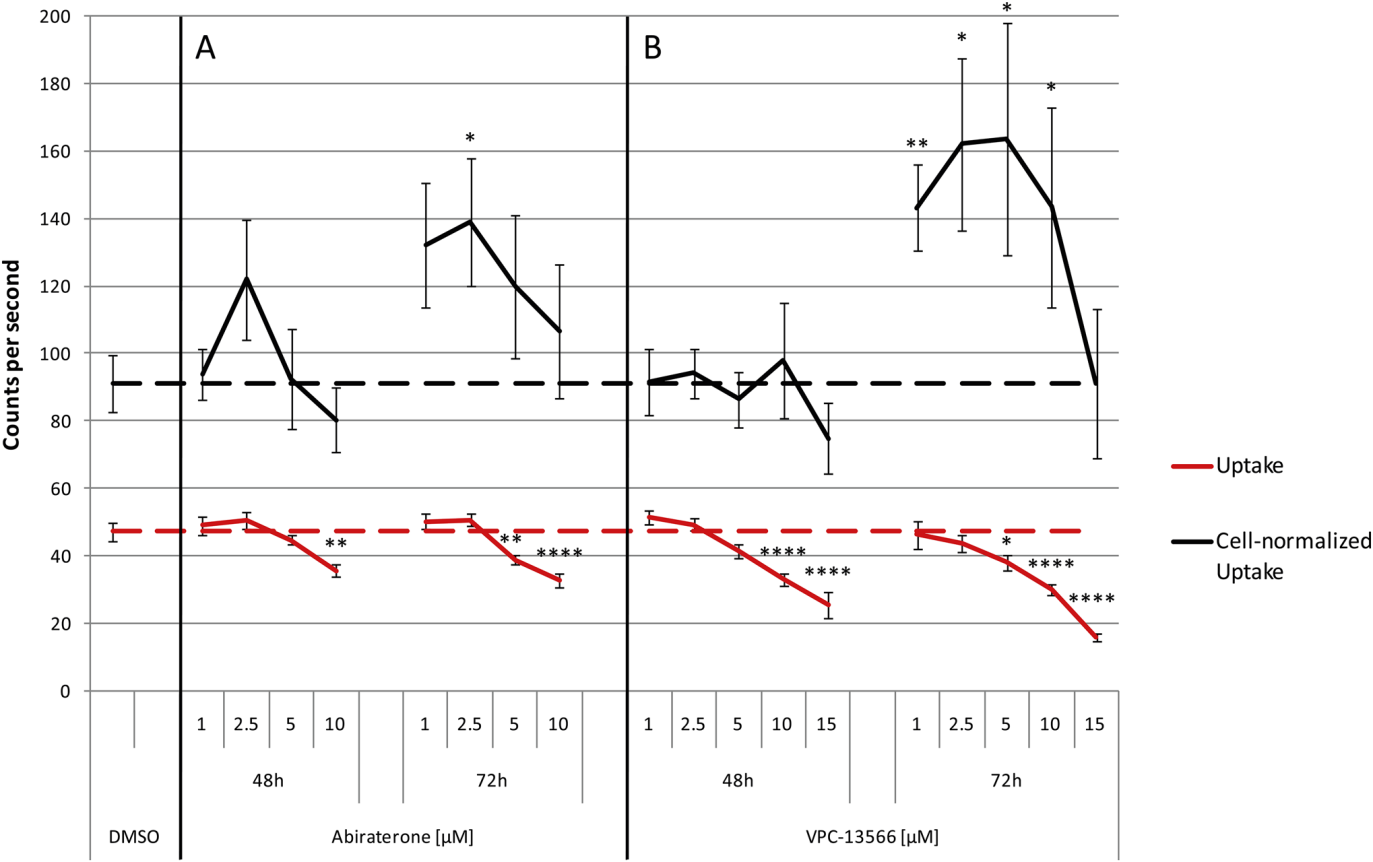
Uptake of [^68^Ga]Ga-PSMA-11 in LnCaP cells treated with abiraterone or VPC-13566. Uptake and uptake normalized to cell number of [^68^Ga]Ga-PSMA-11 in LnCaP cells treated for 48 h, respectively, 72 h, with either DMSO (reference lines) or increasing concentrations of anti-hormones **a** abiraterone acetate or **b** VPC-13566 (1, 2.5, 5 and 10 µM for both and 15 µM for VPC-13566).Values presented are counts per second (mean ± SEM) of *n* = 10 experiments. *: = *p* < 0.05, **: = *p* < 0.01, ****: = *p* < 0.0001

Our data indicate that abiraterone or VPC-13566 treatment increases PSMA expression in LNCaP cells. Therefore, binding of PSMA ligands, such as [^68^Ga]Ga-PSMA-11, should also be increased by this treatment. As the change in PSMA expression due to VPC-13566 occurred most prominent at concentrations higher than 1 µM, we studied a concentration range from 1 to 15 µM for uptake measurements. To test the effect of abiraterone, we used the entire concentration range studied for modulation of PSMA protein expression. Both abiraterone (Fig. 5a) and VPC-13566 (Fig. 5b) significantly reduced total uptake after 90 min of incubation with the tracer at anti-hormone concentrations higher than 5 µM (*p* = 0.002 to *p* = 1.40 × 10^–5^ for treatment with 10 µM abiraterone or VPC-13566 for 48 or 72 h). The decrease in uptake was concentration- and time-dependent and most pronounced after 72 h of incubation. VPC-13566 was comparably active at a concentration of 10 µM. Reduced binding of [^68^Ga]Ga-PSMA-11 by LNCaP cells upon stimulation with anti-hormones does not match our observation that PSMA protein expression is upregulated under these conditions. As both anti-hormones significantly block growth of LNCaP cells, we normalized uptake values to cell count. Normalized uptake values were significantly increased after 72 h of treatment with abiraterone at a concentration of 2.5 µM (*p* = 0.016). At higher abiraterone concentrations, no significant increase was detectable. In contrast, VPC-13566 showed a significantly increased cell-specific uptake in the concentration range between 1 and 10 µM (*p* = 0.002 to *p* = 0.049). Concentrations higher than 15 µM did not increase cell-specific uptake.

For all experiments, PC3 cells served as negative control and exhibited as expected no relevant FOLH1 and KLK3 expression, no detectable PSMA protein expression and PSA secretion, as well as less than 5% of overall uptake of [^68^Ga]Ga-PSMA-11 compared to LNCaP cells (data not shown).

## Discussion

In the present study, we investigated the effect of VPC-13566 and abiraterone on PSMA expression and [^68^Ga]Ga-PSMA-11 uptake. We compared these results to the effects of the two therapeutics on PSA secretion and expression. Our results indicate that PSMA expression and PSA secretion are independently and inversely regulated in LNCaP prostate cancer cells after treatment with anti-hormones. VPC-13566 and abiraterone block PSA secretion consistent with the role of PSA measurement in monitoring anti-hormone treatment of prostate cancer patients. By measuring [^68^Ga]Ga-PSMA-11 uptake in total monolayers of LNCaP cells, we observed downregulation of PSMA protein due to reduced cell growth after treatment with VPC-13566 or abiraterone, whereas the PSMA protein amount per cell was upregulated under these conditions. These findings explain why, in many cases, PSMA PET and PSA values do not match after antihormonal treatment. This may be a temporary discordance during the period when anti-hormones already act on tumor cells, but cell numbers are not yet reduced.

Although the uptake experiments were carried out exclusively with [^68^Ga]Ga-PSMA-11, we assume that at least the general trends of our results can be transferred to the uptake behavior under anti-hormone treatment of other PSMA-affine tracers based on the urea-binding motif, namely the PET tracer [^18^F]F-PSMA-1007 and the therapy tracer [^177^Lu]Lu-PSMA-617. The assumption is based on the following findings: first, the specific tracer uptake in LNCaP tumors in mice models of the tracers [^68^Ga]Ga-PSMA-11, [^18^F]F-PSMA-1007 and [^177^Lu]Lu-PSMA-617 agrees within the error limits 1 h post injection (7.70 ± 1.45%ID/g, 8.04 ± 2.39%ID/g, 11.20 ± 4.17%ID/g, respectively), albeit the organ distributions apart from the tumor differ to some extent (Eder et al. 2012; Benešová et al. 2015; Cardinale et al. 2017). However, the PSMA expression in other organs is probably less anti-hormone-dependent than the tumor itself. Furthermore, very similar uptake and internalization of [^68^Ga]Ga-PSMA-11 and [^177^Lu]Lu-PSMA-617 were demonstrated by PC3-PIP cells (Umbricht et al. 2017), this finding was also used in the reasoning by Lückerath et al. (2018) to transfer the tumor uptake measured by means of [^68^Ga]Ga-PSMA-11 on [^177^Lu]Lu-PSMA-617. In addition, Benesova et al., found that the specific tumor uptake of [^68^Ga]Ga-PSMA-11 and [^177^Lu]Lu-PSMA-617 as well as the organ distribution were very similar (Benešová et al. 2015), indicating that different radionuclides have no significant effect in this context, as long as the tracer structure is the same, in accordance with the general concept of theranostics. Ultimately, by determining the expression of the target protein PSMA, we were able to show that the changes in the cell-specific uptake of [^68^Ga]Ga-PSMA-11 were caused by changes of the target protein expression which are tracer-independent.

A complex relationship between androgen blockade and PSMA expression and PSA secretion has been found previously by in vitro studies and in clinical reports (Wright et al. 1996; Evans et al. 2011; Miyamoto et al. 2012; Paller et al. 2019). Uptake of PSMA-binding tracers was stimulated by androgen deprivation in three different cell lines. Androgen deprivation has different effects on PSMA expression and PSA secretion in patients with hormone-sensitive or castration-resistant prostate cancer. In men with hormone-sensitive cancers, androgen deprivation leads in most cases to a decrease in PSMA-PET maximum standardized uptake values (SUV_max_) and mean standardized uptake values (SUV_mean_) as well as PSA secretion. In men with castration-resistant prostate cancer, androgen deprivation leads to increased SUVs in PSMA PET, but PSA response was delayed or even increased (Emmett et al. 2019). Therefore, it can be speculated that androgen deprivation also enhances uptake of PSMA-based radiopharmaceuticals, such as [^177^Lu]Lu-PSMA-617, and thereby facilitates therapeutic efficacy. To date, this hypothesis has not been confirmed in clinical trials. Our pre-clinical data indicate that, on the one hand, VPC-13566 increases PSMA tracer uptake per cell, while on the other hand, it reduces the cell proliferation. Therefore, under these conditions, SUV in tumor lesions may decrease due to a lower amount of target protein despite the fact that PSMA expression per cell is higher. Admittedly, an increase in the uptake of [^68^Ga]Ga-PSMA-11 by enzalutamide does not necessarily lead to a measurable increase in radio-ligand therapy success in vivo (Lückerath et al. 2018). The question thus arises whether PSMA induction by enzalutamide alone is sufficient to facilitate the therapeutic effect. PSMA induction, however, may be further enhanced by a combination of anti-androgens with different mechanism of action, such as enzalutamide and VPC-13566. By means of the serum biomarker PSA, the combination was already successfully tested (Lallous et al. 2016). Our results are in line with a large body of biochemical evidence. A number of previous studies have shown modulation of PSMA expression by blocking the androgen receptor and its natural ligands testosterone and diydrotestosterone. Reduction of steroid concentration in serum by charcoal stripping induced elevated PSMA-mRNA expression in LNCaP cells (Israeli et al. 1993). This was later confirmed at the protein level by ELISA (Wright et al. 1996). Evans et al. were the first to demonstrate an increase of PSMA expression by treatment with the androgen receptor inhibitor enzalutamide (Evans et al. 2011). In the same study, increased PSMA expression due to enzalutamide treatment was confirmed in vivo by PET imaging with a ^64^Cu-labeled J591 antibody. An increasing PSMA expression on the cell surface has also been demonstrated by flow cytometry for abiraterone and enzalutamide (Murga et al. 2015). Thereafter, Meller et al. (2015) proved an increased uptake of [^68^Ga]Ga-PSMA-11 in VCaP cells treated over 20 passages with abiraterone and for testosterone sensitive subtype after testosterone withdrawal combined with abiraterone addition. Kranzbühler et al. (2018, 2019) expanded the spectrum with the 5α-reductase inhibitor dutasteride, which blocks metabolism of testosterone to dihydrotestosterone by testing PSMA total protein expression, surface expression and uptake of [^177^Lu]Lu-PSMA-617 time- and concentration-dependently. First results in humans were obtained by Hope et al. (2017), when a seven-fold increase of PSMA after treatment with leuprolide acetate and bicalutamide of castration-sensitive carcinoma was reported by [^68^Ga]Ga-PSMA-11-PET. Inverse effects were shown by a similar treatment regime also in castration-sensitive carcinoma by Emmett et al. in (2019). However, these authors stated an increase of PSMA in PET imaging in castration-resistant carcinoma treated with enzalutamide or abiraterone. Prospective human data on treatment outcome after combination of androgen signaling inhibition together with [^177^Lu]Lu-PSMA-617 therapy are not yet available. As already mentioned, a mouse xenograft study with LNCaP C4-2 tumors was in fact unable to demonstrate evidence for higher treatment efficacy of the combination compared to single radio-ligand therapy, although a higher uptake was assumed after enzalutamide addition (Lückerath et al. 2018). All these results are in line with the findings of our study.

## Conclusion

In summary, our data indicate that anti-androgens, such as VPC-13566, regulate PSA secretion on the one hand, and PSMA expression/PSMA tracer binding on the other hand, in the opposite direction. This might have to be considered when PSMA PET is used for therapy monitoring, but needs further validation in in vivo settings.
